# Alleviation of drought stress in pulse crops with ACC deaminase producing rhizobacteria isolated from acidic soil of Northeast India

**DOI:** 10.1038/s41598-018-21921-w

**Published:** 2018-02-23

**Authors:** Juthika Saikia, Rupak K. Sarma, Rajashree Dhandia, Archana Yadav, Rupjyoti Bharali, Vijai K. Gupta, Ratul Saikia

**Affiliations:** 1 Biotechnology Group, Biological Sciences and Technology Division, CSIR-North East Institute of Science and Technology, Jorhat, 785006 Assam India; 2 Department of Biotechnology, Gauhati University, Guwahati, 781014 Assam India; 3 Department of Chemistry and Biotechnology, ERA Chair of Green Chemistry, Tallinn University of Technology, Tallinn, 12618 Estonia

## Abstract

The agricultural crops are often affected by the scarcity of fresh water. Seasonal drought is a major constraint on Northeast Indian agriculture. Almost 80% of the agricultural land in this region is acidic and facing severe drought during the winter period. Apart from classical breeding and transgenic approaches, the application of plant-growth-promoting bacteria (PGPB) is an alternative strategy for improving plant fitness under stressful conditions. The 1-aminocyclopropane-1-carboxylate (ACC) deaminase-producing PGPB offer drought stress tolerance by regulating plant ethylene levels. The aim of the present study was to evaluate the consortium effect of three ACC-deaminase producing rhizobacteria – *Ochrobactrum pseudogrignonense*RJ12, *Pseudomonas* sp.RJ15 and *Bacillus subtilis*RJ46 on drought stress alleviation in *Vigna mungo* L. and *Pisum sativum* L. Consortium treatment significantly increase seed germination percentage, root length, shoot length, and dry weight of treated plants. An elevated production of reactive oxygen species scavenging enzymes and cellular osmolytes; higher leaf chlorophyll content; increase in relative water content and root recovery intension were observed after consortium treatment in comparison with the uninoculated plants under drought conditions. The consortium treatment decreased the ACC accumulation and down-regulated ACC-oxidase gene expression. This consortium could be an effective bio-formulator for crop health improvement in drought-affected acidic agricultural fields.

## Introduction

Climate change is the greatest threat to world’s agricultural sustainability in the 21^st^ century^1^. Drastic changes in various climatic factors (e.g., precipitation, heat, light, etc.) can tremendously influence the global reduction in crop yields^2^. The improvement in crop yields under unfavourable conditions by classical breeding or gene transfer techniques pose certain limitations in terms of ethical issues and time requirements^3^. Again, drought stress tolerance is often a complex phenomenon involving clusters of gene networks^4–6^. Although many of the networks are resolved, a large gap still remains^7^. The inadequate resolution of the diverse gene networks among large numbers of cultivars of a single crop is another serious problem in developing stress-resistant varieties by utilizing the gene technology approach^8^. Therefore, alternative eco-friendly approaches are much more appreciable at this time. One such strategy could be the use of stress-resistant plant growth promoting bacteria (PGPB) with critical roles in enhancing plant growth performance under stressed environments. PGPB either thrive freely in the soil or colonize the rhizosphere, phyllosphere, or plant tissue interior (endophytes). They are already being used as an efficient candidate to improve plant growth and development during normal as well as during stressful environmental conditions^4^. PGPB are capable of producing different plant growth hormones (auxin, gibberellins, cytokinins, and ethylene) and other growth-enhancing molecules (siderophore, hydrogen cyanide, phosphatase, nitrogenise, etc.) which have potential impacts on plant growth and development under abiotic stresses^9^. Under ambient conditions, ethylene confers a beneficial effect on plant health; however, an abrupt increase in ethylene production during biotic and abiotic stresses has negative effects, too, which leads to senescence^10,11^. Interestingly, some PGPB strains with ACC deaminase activity can lessen the inhibitory effects of ethylene in stress-inflicted plants by cleaving the ethylene ACC into α-ketobutyrate and ammonia^10^. Under abiotic stress, rhizobacteria with ACC deaminase activity can improve plant growth and development by regulating ethylene synthesis^10,12,13^. There are many other reports on plant health improvement through inoculating ACC deaminase positive bacterial strains during droughts^14,15^, flooding stress^16^, excessive salinity^17,18^, heavy metal stress^19,20^ etc. However, some of the investigations did not directly describe the causality of the bacterial ACC deaminase enzyme in stress resistance. There might exist some other bacterial determinants for stress alleviation in affected plants. Plant-hormone-related bacterial traits, such as the regulation of indole acetic acid (IAA) levels, also have a distinct role in eliciting stress tolerance in host plants^21^. Recently, Ledger *et al*. reported the role of volatile compounds of ACC deaminase mutant *P. phytofirmans* PsJN strains in inducing salinity resistance in *Arabidopsis* thaliana^22^. Thus, screening ACC deaminase producing strains, as well as different plant-growth-promoting (PGP) traits, to ameliorate abiotic stresses seems to be of greater importance for stressed agricultural systems.

In tropical countries, drought has been identified the main constraint leading to the reductions in crop yields^8^. During stress conditions, the plant-water relation at the cellular level gets destabilized, thus affecting the whole plant^23^. However, plants respond to water shortages with several morphological and physiological modifications^24^. Stress-tolerant PGPB can play a crucial role in these modifications and helps the plants survive. Changes in root architecture (i.e., root system topology and spatial distribution of primary and lateral roots) are the most important adaptive measures in plants during a drought. However, PGPB treatments promote root growth and alter root architecture, leading to an increase in root surface area and improved water and nutrient uptake^25^. Similarly, PGPB inoculation could maintain shoot growth at near-normal levels, resulting in improvements in crop health and productivity^15^. Again, there is a direct correlation between increases in plant relative water content and PGPB treatment. Casanovas *et al*. (2002) reported a positive correlation between bacterialabscisic acid (ABA) production and RWC content in maize plants, which thereby induces stomata closure when inoculated with *Azospirillum brasilense* BR11005spp^26^. Cellular osmotic adjustment by increased content of cellular osmotica is another key adaptation in plants during a drought. PGPB treatments lead to increases in plant cellular osmolytes and help plants to withstand stress^15,27,28^. In severely drought-stressed plants, free radical accumulation leads to the damage of cell membranes and other cellular machinery^29^. Antioxidant enzymes, like catalase (CAT) and peroxidase (POD), have the ability to eliminate free radicals and prevent cell membranes and DNA content from further damage^30^. Certain PGPB can raise the levels of reactive oxygen species (ROS) scavenging enzymes in plants. For instance, Kohler *et al*. reported high antioxidant enzyme activity in lettuce plants (*Lactuca sativa* L.) inoculated with *Pseudomonas mendocina* and *Glomus intraradices*, which contribute to enhancing tolerance against drought^28^ Figueiredo *et al*. detected enhanced antioxidant enzymatic activity in common bean plants (*Phaseolus vulgaris* L.) coinoculated with *Rhizobium tropici* and *Paenibacillus polymyxa* under drought stress conditions^31^. Furthermore, ROS scavenging enzymatic activity was increased in green gram plants (*Vigna radiata* L.) that were inoculated with *Pseudomonas fluorescens*, *Bacillus subtilis and Pseudomonas* aeruginosa^15,27^. In another study, the *Pseudomonas putida* strain GAP-P45, which has the ability to produce exopolyschharides (EPS), alleviated drought stress in sunflower (*Helianthus annuus* L.) seedlings by activating the host plant’s antioxidant enzyme machinery^32^.

Henceforth, inoculation of bacterial isolates that are able to alleviate drought stress could be preferable in the context of environmentally sustainable agriculture. Considerable progress in this context has been made worldwide. However, very little has been done in Northeast India, despite its rich biodiversity in the Indo-Burma Mega hotspot zone^33^. Nearly, 80% of agricultural lands in Northeast India are acidic due to Al^3+^ toxicity^34,35^. Also, although it is known as high rainfall area, these lands experience severe water scarcity during the winter season^34,35^. Increases in Al^3+^ toxicity and subsequent drought stress are resulting in root growth retardation, leading to fewer uptakes of water and nutrients and, thereby, lower crop productivity^35^. However, the alkaline/acidic environments sustain a diverse microbial community with PGP attributes^36^. Previously, few acidotolerant bacterial genera have been found in acidic environments and have sorted out their PGP attributes in low pH conditions^37,38^. Similarly, the acidic soil of north-east India may harbour rich microbial communities that might be useful in agriculture. Previously, our group did substantial work on fluorescent pseudomonads mediated drought stress tolerance in mung beans (*Vigna radiata* L.)^15^. A comprehensive work based on physiological and molecular approaches have established *Pseudomonas aeruginosa* GGRJ21 as a very efficient osmotic stress tolerant strain, having the attributes to enhance drought stress tolerance in host plants. Thus, in a continuation of the previous work, the present investigation focused on screening potent ACC deaminase producers from drought-prone agricultural fields of this region and their effect on drought stress alleviation. Black gram (*Vigna mungo* L.) and the garden pea (*Pisum sativum* L.) were used as model plants. The experimental plants were selected based on their wide cultivation in the sampling sites. The seasonal drought in the winter season has a tremendous negative effect on their growth and production^39^. The rhizosphere bacterial strains were screened for osmotic stress tolerance and ACC deaminase activity for plant growth promotion. Possible inherent mechanisms of drought tolerance were investigated by determining the accumulation of ROS scavenging enzymes (e.g., CAT, POD) and osmolytes (as proline and total phenolics) in the bacterial consortium inoculated plants under water stress conditions. Furthermore, the accumulation of ACC in tested plants and a preliminary investigation on the possible molecular mechanism of bacterial ACC deaminase action on inoculated plants was conducted by examining the expression level of ACC synthase (*ACS*) and ACC oxidase (*ACO*), coding mRNA transcripts by real-time qPCR.

## Results

### Identification, characterization and *in vitro* plant growth promoting traits of selected bacterial isolates

The selected isolates were identified based on their morphological, biochemical, and molecular characteristics. The cells of RJ12 were gram-negative and non-spore forming. Gram-negative rod-shaped cells of RJ15 did not produce spores. However, the cells of RJ46 were found to be gram-positive, spore-forming and rod-shaped. RJ12 showed positive activity for catalase, oxidase, and methyl red test but showed negativity in starch hydrolysis and nitrate reduction. RJ15 was positive for oxidase, catalase, and nitrate reductase but was negative for starch hydrolysis and methyl red test. RJ46 was found to be positive for catalase, oxidase, nitrate reductase and starch hydrolysis but had negative activity in methyl red. Further, the isolates RJ12, RJ15 and RJ46 were identified as *Ochrobactrum pseudogrignonense* (KM271984), *Pseudomonas* sp. (KJ801950) and *Bacillus subtilis* (KM083797), respectively, based on their 16S rRNA gene sequence homology.

The *in vitro* multiple PGP traits of the selected osmotic stress resistant strains, *viz* RJ12, RJ15 and RJ46 were detected in normal and osmotic stress conditions (Table 1). A comparison of all the three strains with the osmotic stress susceptible strain *Serratia nematodiphila* RJ10 revealed a significant effect of osmotic stress on PGP traits. The quantitative estimation showed a significant reduction (*p* = 0.05) in PGP traits, except for ACC deaminase, in all the bacterial strains in osmotic stress condition. However, the reduction rate was lower in RJ12, RJ15 and RJ46 than in RJ10. The isolate RJ12 was recorded as the most efficient ACC deaminase producer, even under osmotic stress conditions (114 nmol mg^−1^ h^−1^), followed by RJ46 (110 nmol mg^−1^h^−1^) and RJ15 (46 nmol mg^−1^h^−1^). Also, RJ12 showed superior activity in other PGPR traits during normal and osmotic stress conditions. All the strains could produce IAA (68–85 µg ml^−1^), phosphatase (37.3–42.6 µg ml^−1^), siderophore (6.2–11.32 μmol benzoic acid ml^−1^) and HCN (9.4–14.3 nmol mg cellular protein^−1^) during osmotic stress conditions. Compatibility assay among the three strains did not show any antagonistic effects against one another (Supplementary Material Figure S1).

**Table 1:** Plant growth promoting traits of bacterial strains, *Ochrobactrum pseudogrignonense* RJ12, *Pseudomonas* sp RJ15 and *Bacillus subtilis* RJ46 under normal and osmotic stress condition (−0.73 MPa). An osmotic stress sensitive PGP strain *Serratia nematodiphila* RJ10 was taken for comparison throughout the experiment.

Bacteria strain	ACC deaminase (n mol mg^−1^ h^−1^)	IAA (µg ml^−1^)at 100 µg ml^−1^ tryptophan	Phosphate Solubilisation index (µg ml^−1^)	Siderophore production μmol benzoic acid ml^−1^	HCN production (nmole mg cellular protein^−1^)	Nitrogen fixation
**Bacterial growth under normal condition**						
*Ochrobactrum pseudogrignonense* J12	125 ± 2.14a	120.9 ± 1.4a	85.4 ± 0.93a	21.76 ± 1.4a	36.2 ± 1.2a	+
*Pseudomonas* sp. RJ15	57 ± 1.06b	100.7 ± 1.5b	69.2 ± 0.88b	14 ± 0.35b	26.7 ± 0.9b	+
*Bacillus subtilis* RJ46	116 ± 1.21c	75.1 ± 0.86c	82.1 ± 1.12a	18.3 ± 0.92a	30.3 ± 0.9c	+
*Serratia nematodiphila* RJ10	132 ± 1.24a	117.3 ± 1.3a	86 ± 0.24a	26 ± 1.1c	38.4 ± 1.2a	+
**Bacterial growth under osmotic stress condition** (**−0.73 MPa**)						
*Ochrobactrum pseudogrignonense* RJ12	110 ± 1.87a	85 ± 1a	42.6 ± 0.73a	11.32 ± 0.2a	14.3 ± 0.7a	+
*Pseudomonas* sp. RJ15	46 ± 0.87b	72 ± 1.12b	37.3 ± 0.54b	6.2 ± 0.11a	9.4 ± 0.2a	+
*Bacillus subtilis* RJ46	114 ± 1.54a	68 ± 0.91c	39.2 ± 0.42b	9.43 ± 0.4a	11.5 ± 0.3a	+
*Serratia nematodiphila* RJ10	4.1 ± 0.41c	10.3 ± 0.51d	3.2 ± 0.01c	1.4 ± 0.14b	2.2 ± 0.14b	−

One-way ANOVA was performed in both the table considering the activity of *Serratia nematodiphila* strain RJ10 as independent variable, followed by Tukey’s test. Means within a column sharing same lowercase letter are not significantly different at *p* = 0.05; figures are means ± standard deviation (n = 5). Table key: “+” positive for the test; “−” negative for the test.

### Bacterial effect on seed germination and plant growth promotion

Consortium treatment had a significant effect on seed germination and vigor index compared to individual bacterial treatment or a random combination of any of the two bacterial strains in stress condition (Table 2). Seed treatment with consortium resulted in 100% germination in both test plants. A higher vigor index in both black gram (vigor index_control_ = 1325, vigor index_consortium_ = 3100) and garden pea (vigor index_control_ = 1328, vigor index_contortium_ = 2870) plants clearly showed a significant (*p* = 0.05) increase in root length and shoot length in germinated seedlings treated with bacterial consortium compared to a combination of any two isolates or individual bacterial treatments (Table 2).

**Table 2:** Effect of bacterial inoculation on growth attributes of black gram and garden pea. (RJ12 - *Ochrobactrum pseudogrignonense*, RJ15 - *Pseudomonas* sp, RJ46 - *Bacillus subtilis*).

Treatments	Germination (%)		Root length (cm)		Shoot length (cm)		Vigor index	
	Black gram	Garden pea	Black gram	Garden pea	Black gram	Garden pea	Black gram	Garden pea
Un-inoculated seeds	87 ± 1b	83 ± 4.7c	4.1 ± 0.76a	3 ± 0.34e	11 ± 1.3b	13 ± 1.1b	1325 f	1328 f
RJ12 inoculated seeds	98 ± 2.3a	97 ± 4.7a	7.5 ± 1b	6 ± 0.82d	17.1 ± 2.1a	15.7 ± 1.5a	2410b	2104c
RJ15 inoculated seeds	97 ± 4.7a	90 ± 8b	5.2 ± 0.87a	5.8 ± 0.9d	16.7 ± 1.7a	16 ± 1.7a	2215d	1962e
RJ46 inoculated seeds	90 ± 1.7b	93 ± 4.7b	6 ± 0.45b	7 ± 0.23c	16 ± 1.8a	16.2 ± 1.6a	1980e	2157c
RJ12 + RJ15 inoculated seeds	96 ± 1.6a	94 ± 5b	8.3 ± 1.2b	7 ± 0.64c	15.8 ± 2.01a	15 ± 1.0a	2313c	2068d
RJ12 + RJ46 inoculated seeds	98 ± 2.5a	97 ± 3a	9.1 ± 1b	8.5 ± 1b	16.2 ± 1.3a	16.4 ± 2.0a	2479b	2415b
RJ15 + RJ46 inoculated seeds	94 ± 1.9b	96 ± 4.3a	8 ± 1.5b	7.3 ± 0.56c	15 ± 2.0a	15.7 ± 1.45a	2162d	2208c
Consortium treated seeds	100 ± 0a	100 ± 0a	14 ± 1a	12 ± 1a	17 ± 1.5a	16.7 ± 1.0a	3100a	2870a

Means within a column sharing same lowercase letter are not significantly different according to Student’s t-test at *p* = 0.05; figures are means ± standard deviation (n = 5).

### Pot study under water stress

**Figure 1:**
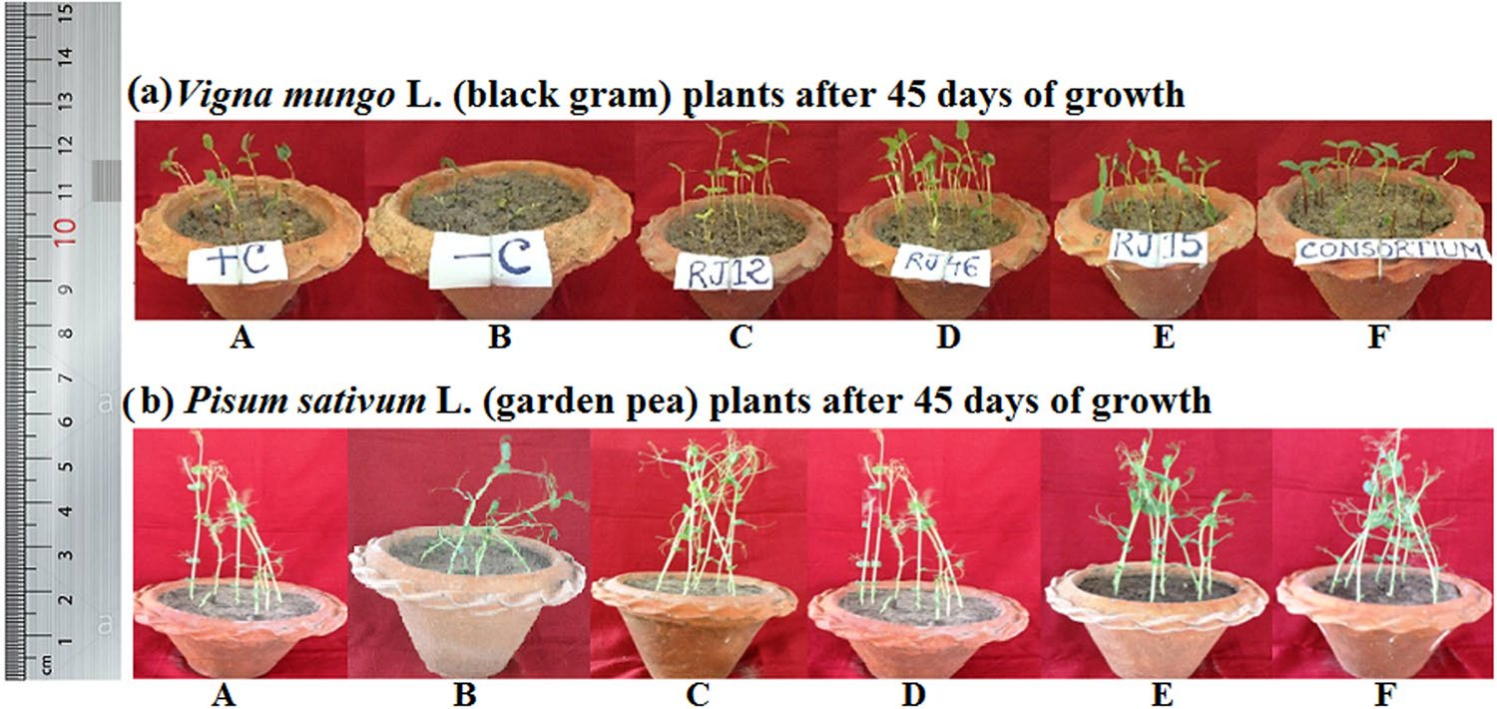
Effect of bacteria inoculation on plant growth promotion under water stress (drought). Bacterial individual and combined effect (consortium) increases survival rate of (**a**) black gram and (**b**) garden pea plants after 45^th^ days of growth stress induction as compared to the negative control (stressed plants without inoculation). A - uninoculated watered plants as positive control, B- uninoculated plants under drought stress as negative control, C – inoculated with *Ochrobactrum pseudogrignonense* RJ12 under water stress, D – inoculated with *Bacillus subtilis* RJ46 under water stress, E – inoculated with *Pseudomonas* sp. RJ15 under drought stress, F – consortium (*Ochrobactrum pseudogrignonense* RJ12 + *Bacillus subtilis* RJ46 + *Pseudomonas* sp. RJ15) inoculated plants with drought stress.

**Figure 2:**
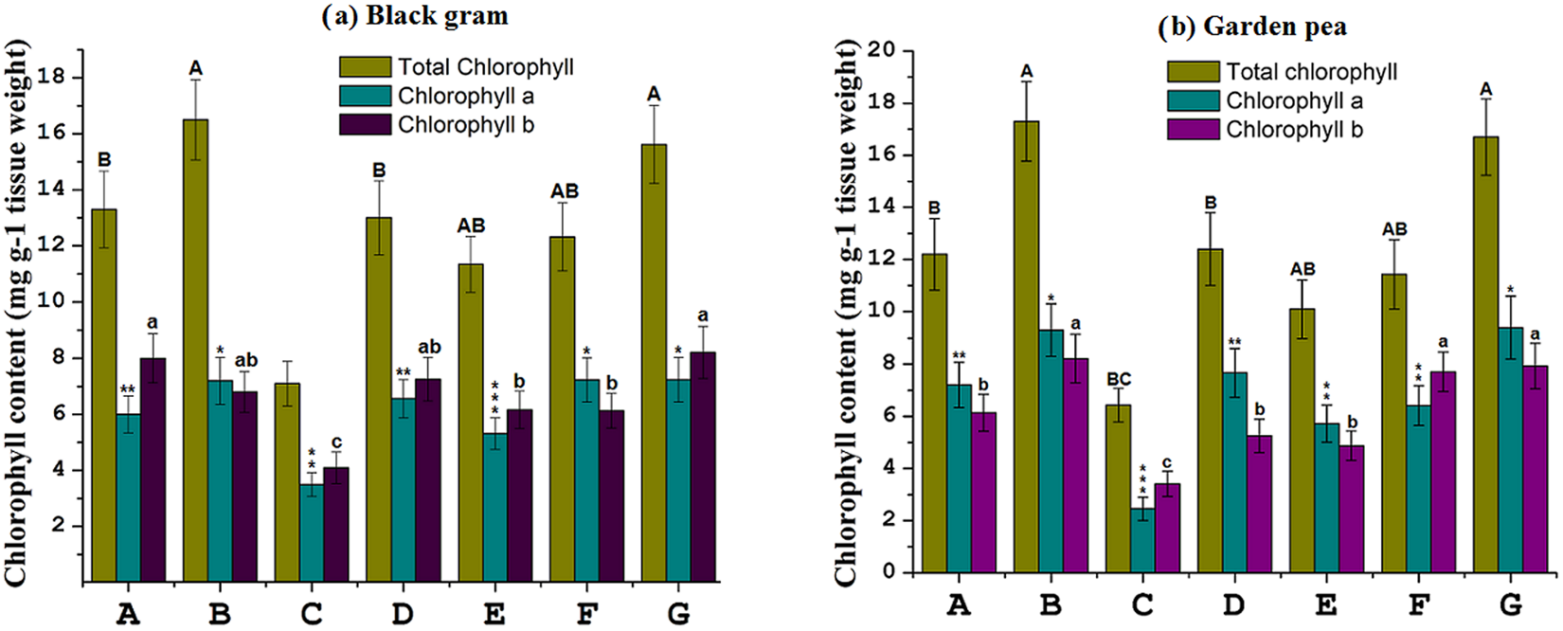
Quantitative estimation of chlorophyll a, chlorophyll b, and total chlorophyll content of (**a**) black gram and (**b**) garden pea leaves growing under different treatment condition. The quantification was carried out after 20 days of stress induction. Treatment conditions, A- positive control, B- consortium (*Ochrobactrum pseudogrignonense* RJ12 + *Bacillus subtilis* RJ46 + *Pseudomonas* sp.RJ15) inoculated with sufficient water supply, C- negative control (stressed plants without inoculation), D-inoculated with *Ochrobactrum pseudogrignonense* RJ12 under stress, E-inoculated with *Pseudomonas* sp.RJ15 under stress, F- inoculated with *Bacillus subtilis* RJ46 under stress, G- consortium (*Ochrobactrum pseudogrignonense* RJ12 + *Bacillus subtilis* RJ46 + *Pseudomonas* sp.RJ15) inoculated under stress. Two-way ANOVA was performed considering water supply and bacteria inoculation as two independent variables followed by Tukey’s post-test for each treatment. Error bars show the standard deviation of the mean values of five replicates. Different symbols on bars indicates a significant difference at *p* = 0.05 in chlorophyll content under different treatment conditions.

The negative effect of water stress was noticed in uninoculated water stress (drought) imposed pulse crops with stunted growth, reduced vigor, and less chlorophyll content (Figs 1 and 2, Table 3). However, significant (*p* = 0.05) plant growth and development was noticed in bacteria-inoculated plants under drought stress (Figs 1 and 2, Table 3, Supplementary Material Table S3). The combined action of the three bacterial strains was found to be more promising than the action of any two combined strains or of each strain used individually (Table 3, Supplementary Material Table S3). The consortium helped the plants to thrive best at the soil moisture content, even at 9.5% (data not shown). At 20% soil moisture content, the consortium treatment increased the root length of black gram and garden pea plants significantly (*p* = 0.05) by 287% and 269%, respectively when compared to the control plants; however, these values in uninoculated stressed plants were 41% and 39% for black gram and garden pea, respectively. Treatment with individual bacterium showed a similar trend, but one that was not superior to their combined action. Consortium treatment did not show any notable difference in shoot length elongation as compared to treatment with individual isolates or a combination of any two of them under stressed conditions. Compared to control plants, 4.2% (black gram) and 17.3% (garden pea) decreased in dry weight in consortium-treated stressed plants. However; this rate for the uninoculated stressed plants was 63.8% and 61.7%, respectively (Table 3). Similarly, remarkable increases of 85% and 88% in RWC (relative water content) was observed in consortium-inoculated stress-imposed black gram and garden pea plants, respectively, as compared to the uninoculated stressed plants. Root recovery intension is one of the most reliable and sensitive indicators of drought tolerance in plants. Consortium treatment was also found to be effective in root recovery intension. Furthermore, the isolates enhanced plant growth promotion in normal irrigated conditions, too (Table 3, Supplementary Material Table S3).

**Table 3:** Effect of bacteria inoculation on plant growth promotion in black gram and garden pea plants (RJ12 - Ochrobactrum pseudogrignonense, RJ15 - Pseudomonas sp, RJ46 - Bacillus subtilis. Consortium is the mixture of all the three strains in equal ratio (1:1:1).

Treatments	Root length (cm)		Shoot length (cm)		Dry weight (g)		Relative water content (%)		Root recovery intension (mg g^−1^ hr^−1)^	
	BG	GP	BG	GP	BG	GP	BG	GP	BG	GP
Uninoculated plants with sufficient water supply	3.12 ± 0.3 f	2.76 ± 0.21 g	5.43 ± 0.42 h	7.54 ± 0.26 g	47 ± 1.9c	65.3 ± 0.76b	62 ± 1 f	73 ± 2.6e	0.76 ± 0.1b	0.79 ± 0.21b
Uninoculated plants under stress	4.4 ± 0.33e	3.86 ± 0.25e	2.52 ± 0.12i	4.0 ± 0.5 h	17 ± 0.23 h	25 ± 0.43i	40 ± 0.65 h	42 ± 0.38i	0.36 ± 0.02e	0.34 ± 0.01 f
Inoculated with RJ12 and sufficient water supply	3.87 ± 0.53 f	3.3 ± 0.13 f	6.3 ± 1.5 g	12.5 ± 1.1e	53 ± 0.54b	67.43 ± 0.4b	78 ± 0.54b	88 ± 0.21b	ND	ND
Inoculated with RJ12 under stress	8.2 ± 0.46b	7.14 ± 0.13b	14.3 ± 0.41b	15.2 ± 0.65c	43 ± 1.7fd	48 ± 0.24 f	68 ± 0.17e	70 ± 0.64 f	0.62 ± 0.14c	0.67 ± 0.27c
Inoculated with RJ15 and sufficient water supply	2.76 ± 0.13 g	2.96 ± 0.76 g	7 ± 1 f	10.7 ± 1.4 f	49 ± 2c	52 ± 1e	68 ± 1.7e	76 ± 1.6d	ND	ND
Inoculated with RJ15 under stress	6.11 ± 0.38d	6.51 ± 0.31d	11.2 ± 0.27d	12.45 ± 0.34e	28 ± 0.27 g	36 ± 0.65 h	61 ± 0.14 f	58 ± 0.61 h	0.51 ± 0.18d	0.64 ± 0.22d
Inoculated with RJ46 and sufficient water supply	3.43 ± 0.23 f	2.8 ± 0.15 g	6.86 ± 0.89 f	11.1 ± 1 f	38 ± 0.54e	57 ± 1.8c	72 ± 2.1d	75 ± 2d	ND	ND
Inoculated with RJ46 under stress	7.1 ± 0.43c	6.82 ± 0.65c	12.67 ± 0.31c	14.3 ± 0.52d	32 ± 0.26 f	41 ± 0.27 g	58 ± 0.32 g	66 ± 0.46 g	0.52 ± 0.16d	0.65 ± 0.13d
Consortium inoculated with sufficient water supply	4.15 ± 0.48e	3.45 ± 0.5 f	16.2 ± 0.28a	17.35 ± 0.43a	62.65 ± 0.65a	87.43 ± 0.32a	87 ± 0.32a	91 ± 0.43a	0.78 ± 0.13a	0.81 ± 0.15a
Inoculated with Consortium under stress	12.1 ± 0.86a	10.2 ± 0.32a	10.73 ± 0.36e	16.71 ± 0.54b	45 ± 0.23d	54 ± 0.32d	74 ± 0.25c	79 ± 0.26c	0.63 ± 0.21c	0.56 ± 3.16e

Two-way ANOVA was performed considering water supply and bacteria inoculation as two independent variables, followed by Tukey’s post-test for each treatment. For each figure in a column, values represented by the same lowercase letters are not significantly different at *p* = 0.05.; figures are means ± standard deviation (n = 5). Table key: BG - black gram, GP - garden pea, ND – not determined.

Leaf chlorophyll content was determined to examine the impact of the rhizobacterial strains as well as their consortium on the photosynthetic efficiency of host plants (Fig. 2). Total leaf chlorophyll content did not bear any significant difference in consortium-treated stressed plants in comparison to uninoculated watered plants (positive control). However, the contents of leaf chlorophylls a, b, and a + b in consortium-treated stressed plants increased by 106%, 100%, 120% (in black gram plants) and 283%, 132%, and 159% (in garden pea plants) in comparison with the uninoculated stressed plants (negative control). This indicates the efficiency of PGPB strains on the maintenance of chlorophyll content under drought conditions.

### Effects on antioxidant enzymes and osmolyte accumulation in plants

**Figure 3:**
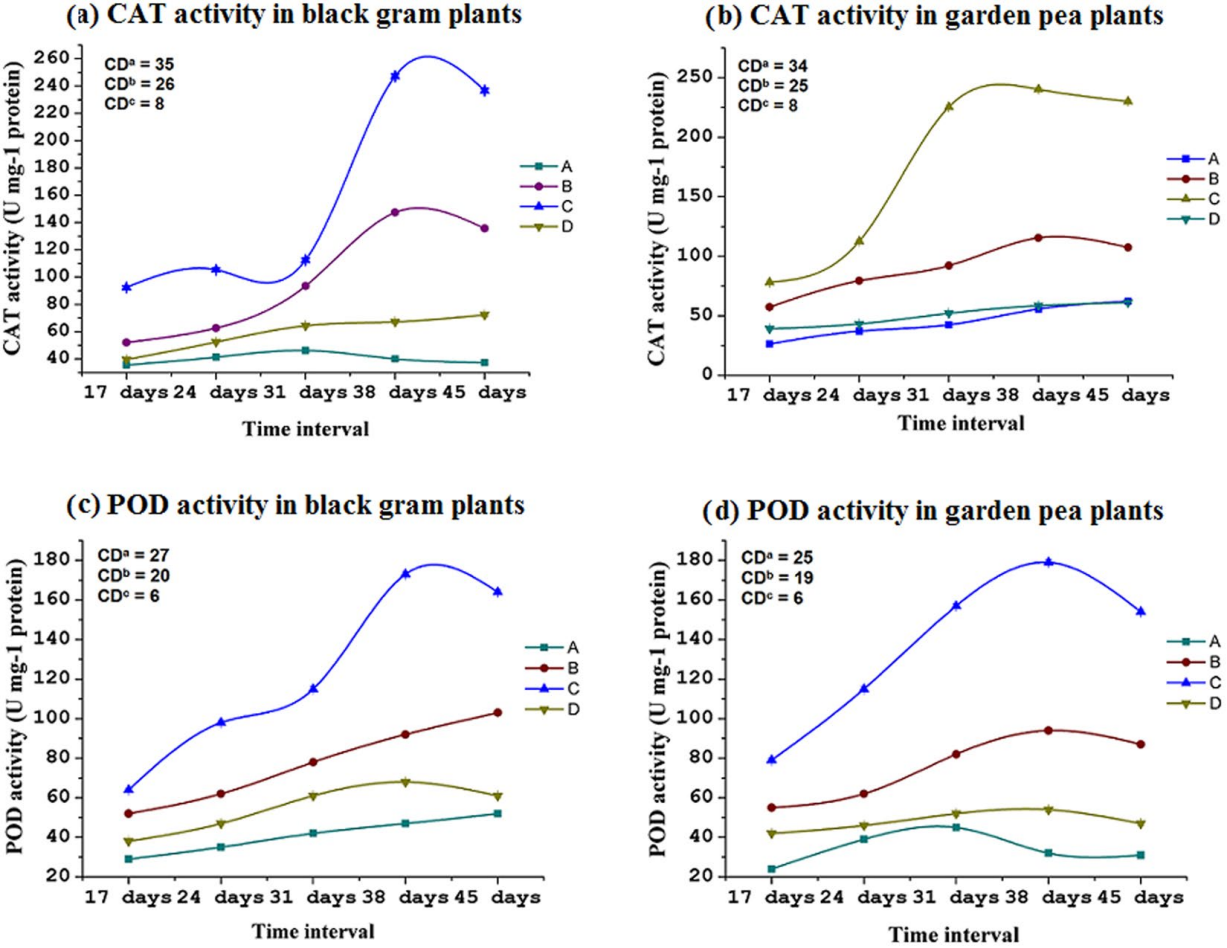
Activity of catalase and peroxidase in stress experienced black gram and garden pea plants in different time interval. Ten samples were analyzed for each replication, and each treatment consisted of five replications. (**a**) catalase activity in black gram, (**b**) catalase activity in garden pea, (**c**) peroxidase activity in black gram, d) peroxidase activity in garden pea. Treatments: A- uninoculated watered plants as positive control, B – consortium (*Ochrobactrum pseudogrignonense* RJ12 + *Bacillus subtilis* RJ46 + *Pseudomonas* sp.RJ15) inoculated plants with drought stress induction, C- uninoculated plants under drought stress as negative control, D – consortium (*Ochrobactrum pseudogrignonense* RJ12 + *Bacillus subtilis* RJ46 + *Pseudomonas* sp.RJ15) inoculated with sufficient water supply. Critical difference (CD) was computed at *p* = 0.05. CDa, CDb, and CDc represent critical differences for treatments, time intervals, and treatment × time intervals, respectively.

The activation of a plant’s inherent enzymatic and non-enzymatic systems is always crucial for the detoxification of the ROS under stress conditions. The results of this study clearly indicated that consortium significantly (*p* = 0.05) stimulates the CAT activity in both black gram and garden pea plants (Fig. 3a,b). CAT is a major enzyme for hydrogen peroxide detoxification in stressed plants. In consortium-treated stressed plants, CAT activity was increased rapidly from the seventeenth day to the thirty-eighth day, after which it declined gradually. Moreover, an increasing trend was also noticed in stress-induced plants without inoculation, but this trend was not as prominent as it was in the consortium-inoculated plants. The control plants and consortium-treated normally-watered plants did not show any significant changes in CAT activity during the experiments. POD was located in every ROS-producing compartment and functions as a fine regulator of intracellular ROS level. As with CAT, a similar increasing trend was also observed in POD activity in consortium-inoculated stress-induced plants as a function of time (Fig. 3c,d). POD activity was gradually increased in the negative control and consortium-treated drought-stressed plants. Furthermore, enzyme activity increased significantly (*p* = 0.05) in consortium-treated plants compared to plants under negative control.

Proline and phenolics are other biomarkers of plants under stress. The increases in proline and phenolic accumulation are very much essential for maintaining the osmotic potential of plant tissues and, thereby, protect plants from over dehydration during droughts. Bacterial inoculation had a direct effect on plant osmolytes (Table 4). Proline content was increased significantly (*p* = 0.05) in consortium-inoculated plants when compared to both positive and negative control plants. However, in consortium-inoculated regularly-watered plants, a marked difference was not observed in comparison to control plants. The leaf phenolics content of bacteria-treated stress-exposed plants was measured from the thirty-eighth day of treatment. Bacterial-combined action in the form of consortium increased the phenolics content by 196% and 216% in black gram and garden pea plants, respectively when compared to the positive control plants. However, these values for uninoculated stress-imposed plants were 97% and 68% in black gram and garden pea, respectively.

**Table 4:** Activity of proline and phenolics in black gram and garden pea plants under water stress condition. Proline activity was measured at different interval of time, i.e. 17^th^ to 45^th^ days of sowing with seven days of interval. Phenolics activity measured on the 38^th^ day of sowing. Consortium is the mixture of all the three strains in equal ratio (1:1:1).

Proline activity (µmoles g^−1^ fresh weight)												
Treatments	Time Interval											
	17^th^ day			24^th^ day		31^st^ day			38^th^ day		45^th^ day	
	BG	GP		BG	GP	BG		GP	BG	GP	BG	GP
Uninoculated plants with sufficient water supply	2.86 ± 0.34c	1.96 ± 0.05c		3.32 ± 0.72c	2.85 ± 0.21c	3.57 ± 0.23c		2.97 ± 0.61c	3.69 ± 0.45c	3.12 ± 0.3b	3.74 ± 0.21c	3.31 ± 0.41c
Uninoculated plants under stress	3.96 ± 0.14b	3.20 ± 4.05b		4.30 ± 1.77b	5.34 ± 0.43a	5.87 ± 0.77b		6.21 ± 0.35b	7.32 ± 0.32b	7.80 ± 0.34a	9.76 ± 0.42b	7.91 ± 0.32b
Consortium inoculated with sufficient water supply	3.65 ± 0.25b	3.12 ± 0.18b		3.98 ± 0.22b	3.45 ± 0.3b	4.12 ± 0.28c		3.56 ± 0.11c	4.19 ± 0.23c	3.76 ± 0.12b	4.31 ± 0.33c	3.89 ± 0.6c
Inoculated with Consortium under stress	4.71 ± 0.26a	4.26 ± 0.54a		5.82 ± 0.81a	5.37 ± 0.31a	8.25 ± 0.12a		7.62 ± 0.18a	9.78 ± 0.54a	8.43 ± 0.42a	12.71 ± 0.34a	10.67 ± 0.55a
**Total phenolic (mg g** ^**−1**^ **fresh weight)**												
**Treatments**			**BG**				**GP**					
Uninoculated plants with sufficient water supply			7.2 ± 1.14c				4.87 ± 0.21c					
Uninoculated plants under stress			14.24 ± 2.21b				8.21 ± 0.15b					
Consortium inoculated with sufficient water supply			8.32 ± 1.1c				5.65 ± 0.14c					
Inoculated with Consortium under stress			21.34 ± 2.12a				15.43 ± 0.52a					

Two-way ANOVA was performed considering water supply and bacteria inoculation as two independent variables, followed by Tukeys post-test for each treatment. For each figure in a column, values represented by the same lowercase letters are not significantly different at *p* = 0.05; figures are means ± standard deviation (n = 5). Table key: BG - black gram, GP - garden pea.

### ACC accumulation in plants

**Figure 4:**
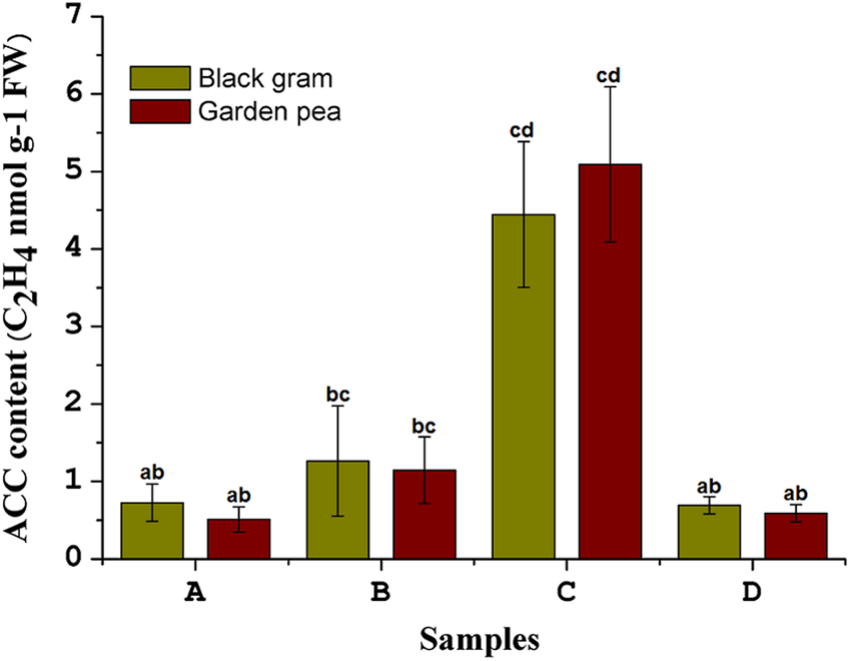
1-aminocyclopropane-1-carboxylic acid (ACC) content in the root tips of black gram and garden pea plants growing under different treatment condition. The quantification was carried out on 45^th^ day of stress induction. Treatments: A- uninoculated watered plants as positive control, B-consortium (*Ochrobactrum pseudogrignonense* RJ12 + *Bacillus subtilis* RJ46 + *Pseudomonas* sp.RJ15) inoculated plants with drought stress induction, C- uninoculated plants under drought stress as negative control, D – consortium (*Ochrobactrum pseudogrignonense* RJ12 + *Bacillus subtilis* RJ46 + *Pseudomonas* sp.RJ15) inoculated with sufficient water supply. Two- way ANOVA was performed considering water supply and bacteria inoculation as two independent variables followed by Tukey’s post-test for each treatment. Different symbols on bars indicates a significant difference at *p* = 0.05 in ACC accumulation under different treatment condition.

Changes in ACC levels in positive control plants, consortium-inoculated stress-induced plants, uninoculated stress-induced plants, and consortium-inoculated regularly-watered plants were further estimated to confirm the positive interaction of ACC deaminase producers with the experimental black gram and garden pea plants. There was significant (*p* = 0.05) reduction in ACC accumulation in consortium-treated stress-induced plants as compared to the uninoculated stress-induced plants (Fig. 4).

### Relative quantification of ethylene synthesis regulatory genes

**Figure 5:**
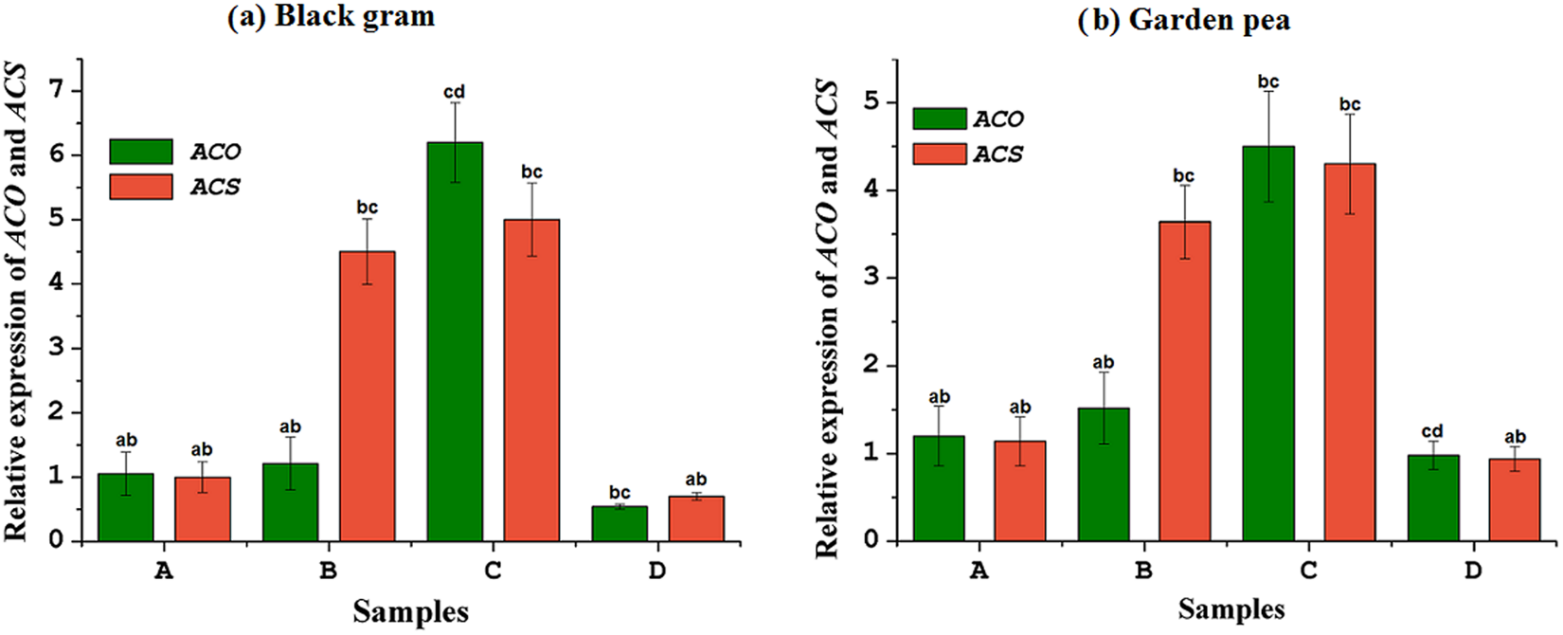
Relative gene expression level of *ACO* and *ACS* in leaf tissue of (**a**) black gram and (**b**) garden pea plants in different treatment conditions. Treatments: A- uninoculated watered plants as positive control, B-consortium (*Ochrobactrum pseudogrignonense* RJ12 + *Bacillus subtilis* RJ46 + *Pseudomonas* sp.RJ15) inoculated plants with drought stress induction, C- uninoculated plants under drought stress as negative control, D-consortium (*Ochrobactrum pseudogrignonense* RJ12 + *Bacillus subtilis* RJ46 + *Pseudomonas* sp.RJ15) inoculated with sufficient water supply. Two-way ANOVA was performed considering water supply and bacteria inoculation as two independent variables followed by Tukey’s post-test for each treatment. Different symbols on bars indicates a significant difference at *p* = 0.05 in gene expression under different treatment condition.

**Figure 6:**
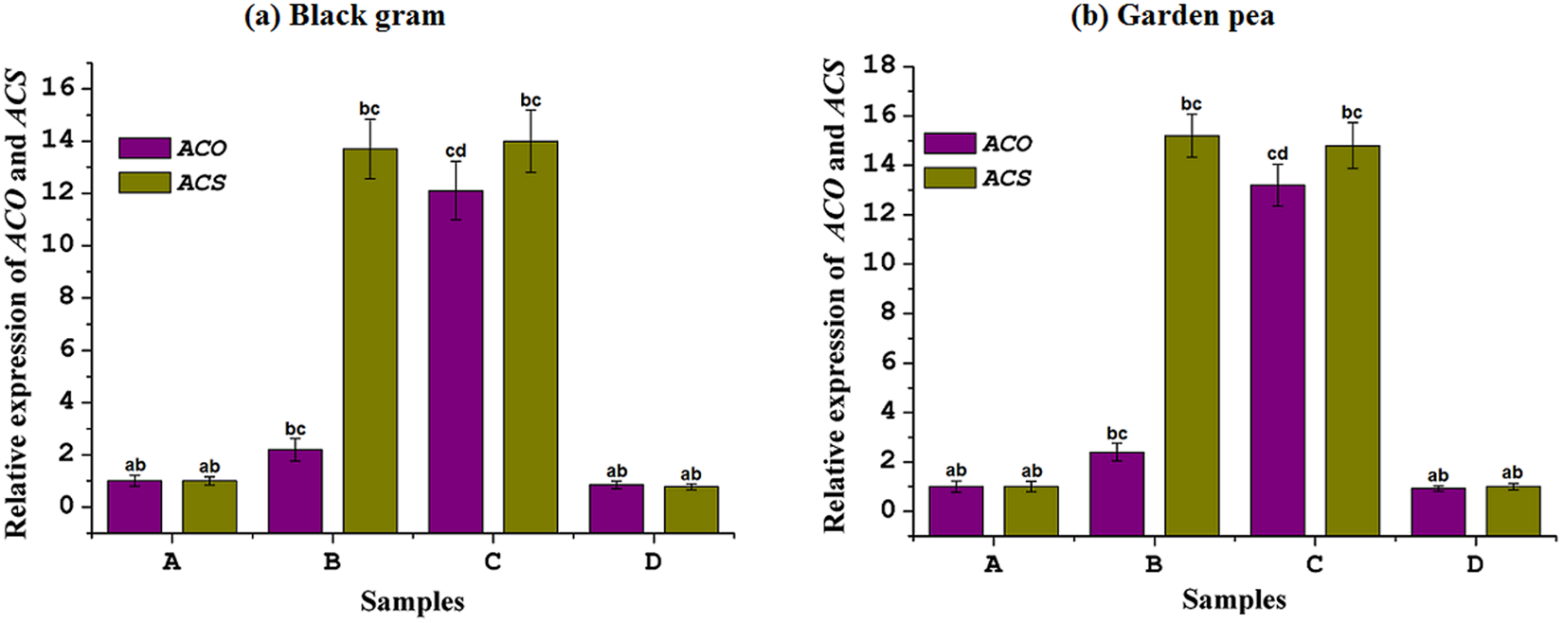
Relative gene expression level of *ACO* and *ACS* in root tissue of (**a**) black gram and (**b**) garden pea plants in different treatment conditions. A- uninoculated watered plants as positive control, B-consortium (*Ochrobactrum pseudogrignonense* RJ12 + *Bacillus subtilis* RJ46 + *Pseudomonas* sp.RJ15) inoculated plants with drought stress induction, C- uninoculated plants under drought stress as negative control, D – consortium (*Ochrobactrum pseudogrignonense* RJ12 + *Bacillus subtilis* RJ46 + *Pseudomonas* sp.RJ15) inoculated with sufficient water supply. Two-way ANOVA was performed considering water supply and bacteria inoculation as two independent variables followed by Tukey’s post-test for each treatment. Different symbols on bars indicates a significant difference at *p* = 0.05 in gene expression under different treatment condition.

Among the four reference genes, *ACT11*, *Ubq*, *β-Tub9*, and 18S rRNA, *ACT11* was verified to have the lowest average expression stability (M) when samples experiencing osmotic stress were analyzed (data not shown). Therefore, *ACT11* was selected as the housekeeping gene for overall expression analysis. The relative expression level of *ACS* and *ACO* genes in consortium-treated black gram and garden pea plants were studied over four experimental conditions: plants with a normal water supply, consortium-inoculated plants with normal water supply, consortium-inoculated plants with induced drought stress, and uninoculated plants under drought stress. The plants with a normal water supply were considered a calibrator for the experiment. Similar to the negative control plants, the transcript copy number of *ACS* was significantly increased in leaf (Fig. 5a,b) and root (Fig. 6a,b) tissues of consortium-treated plants, indicating no significant role of consortium action on the expression of the *ACS* gene transcript. The expression of *ACS* was 8 to 9 times greater in root tissues than in leaf tissues. However, consortium treatment regulated 3 to 5 and 10 times greater down-regulation of *ACO* in leaf and root tissues, respectively, as compared to the uninoculated stress-induced plants (Figs 5a,b and 6a,b).

### Toxicity test

An acute oral toxicity/pathogenicity test was carried out by APT Testing and Research Private Limited (Pune, India) for all three bacterial strains, which were found to be non-toxic according to the EPA 712-C-96–322, OPPTS 8853550 Guidelines (adopted February 1996).

## Discussion

In recent days, water shortage is one of the main challenges faced by worldwide agricultural practices. This issue has been limiting crop yields of arable land^40^. Thus, achieving better crop health and production under water shortage conditions is the biggest challenge for sustainable global agriculture. Again, there is an indirect relationship between soil acidity and reduction in crop yields during drought stress. In general, soil acidity is toxic to plant roots and lead**s** to poor and abnormal root development^35^. This, in turn, leads to reduced water and nutrient uptake. Even a restriction of root penetration into lower soil layers may occur due to increases in acidity of subsoils^41^. Thus, high soil acidity magnifies the negative effect**s** of drought during average rainfall levels, too. In this study, the aim was to identify efficient indigenous osmotic stress tolerant bacterial strains from drought affected acidic agricultural soils of Northeast India for their ability to confer drought resistance in crop plants. Three osmotic stress-tolerant ACC deaminase-producing bacterial strains, (i.e. *Ochrobactrum pseudogrignonense* RJ12, *Pseudomonas* sp. RJ15, and *B. subtilis* RJ46) were screened, and their PGP activities were evaluated in black gram and garden pea plants under water deficit conditions. Huang *et al*. reported that the direct interactions between different microbial members often result in the promotion of key PGP processes and of plant growth and development in a typical rhizosphere ecosystem^42^. Again, the syntrophic relationship between different organisms is quite common in microbial ecosystems^43^. Thus, the use of mixed microbial inoculants (consortium) acting synergistically is more beneficial for better yields and quick/improved results. Moreover, higher concentrations of bacterial cells in a consortium have the potential to supply more nutrients to host plants, thus exerting greater PGP effects^44^. The bacterial strains selected for the present study were compatible with each other. The results of *in-vivo* experiments revealed tremendous improvements in plant growth promotion of test plants under both normal and water deficit conditions when the three strains were applied in consortium compared to individual treatments or mixtures of any two strains.

To validate the effect of ACC deaminase on stress-induced experimental plants, we measured the level of ACC accumulation, as well as mRNA expressions of *ACS* (responsible for ACC production) and *ACO* (responsible for stress ethylene production) gene transcripts in black gram and garden pea plants. Consortium inoculation significantly reduced ACC accumulation in stress-induced black gram and garden pea plant roots when compared to the negative control plants (uninoculated plants under stress). The active ACC deaminase enzyme of bacterial origin might have played a significant role in the reduction of ACC levels in roots, as reported in previous research^45^. Moreover, an up-regulation of *ACS* was recorded in the tested plants in the stress condition. The higher expression of *ACS* may trigger ACC accumulation in plant tissues during the initial phase of stress induction. However, root-or-seed-surface-anchored PGPB with ACC deaminase activity may act as a sink for ACC that lowers their levels in the inoculated plants, as mentioned in previous reports^45,46^. Furthermore, the down-regulation of *ACO* in leaf and root tissues of consortium-inoculated stress-induced plants and the up-regulation of *ACO* mRNA transcripts in negative control plants had strengthened the correlation between bacteria inoculation and reduction in deleterious stress ethylene accumulation in drought-stress-experienced plants. The consortium inoculation might have some effect on *ACO* down-regulation and, thereby, the prevention of deleterious ethylene accumulation in stressed plants. The results are corroboratory with previous findings. Camilios-Neto *et al*. reported the impact of bacterial colonization in *ACO* transcript down-regulation, which leads to a lowering of ethylene accumulation in wheat seedlings during nutrient limitation^47^. Similarly, an excessive down-regulation of *ACO* was noticed in stress-imposed pepper plants (*Capsicum annuum* L.) upon inoculation with PGPB (*Bacillus* sp. and *Arthrobacter* sp.) strains^48^. The lower expression pattern of *ACO* in consortium-treated stressed plants might be due to the substrate-based competition between ACC deaminase and *ACO* for binding with ACC^46^. The reduction in ACC levels would lead to a reduced *ACO* expression and subsequent declines in stress ethylene production. Hence, the bacterial consortium is capable of regulating stress ethylene levels, thereby conferring drought stress tolerance in the tested host plants.

Microorganisms with multi-faceted mechanisms of action are beneficial for plant growth promotion during abiotic stress condition^49^. Therefore, different inherent PGP attributes were also screened and quantified in the selected ACC deaminase positive bacterial strains. The bacterial strains were potent for many PGP traits, such as IAA production, siderophore production, HCN production, phosphate solubilisation, and nitrogen fixation even under high osmotic stress conditions. According to Glick *et al*. (2007), bacterial PGP traits have a positive influence on plant growth and development by increasing nutrient availability during stressful conditions^50^. It is well-known that IAA stimulates the transcription of the plant enzyme ACC synthase^51^, which catalyzes the formation of ACC. In this case, IAA induces ethylene production in the plant. However, increases in ethylene levels have feedback inhibitory effects on IAA signal transduction, which thereby limits the activity of ACC synthase^51^. Thus, the association of IAA-producing bacteria in plants will ultimately trigger the production of relatively high concentrations of ACC and, subsequently, the feedback inhibition of IAA synthesis. However, PGPB with both IAA- and ACC-deaminase-producing activity will control the excess ethylene production level and thereby lessen the ethylene feedback inhibition of IAA biosynthesis. This is because a large portion of the additional ACC produced due to a cumulative effect of plant and bacterial IAA is cleaved by bacterial ACC deaminase. Therefore, the overall result of this cross-talk well defines the role of IAA to enhance plant growth promotion under stressful conditions in the presence of ACC deaminase activity. Previous studies showed the effect of bacterial IAA on root length elongation under a drought stress regime^52,53^. Recently, Sorty *et al*. have reported the influence of bacterial IAA in seed germination and seedling growth in wheat under saline stress^54^. Similar predispositions of bacterial IAA production in seed germination and root length elongation were noticed in our experimental plants. Further, the phosphate-solubilizing bacterial isolates of drought agrosystems are likely to be more useful for plant health improvement under water deficit conditions. Again, the three osmotic stress tolerant strains were not only restricted to the inorganic phosphate bound to calcium ions (i.e. Ca_3_(PO)_4_) but can also act upon aluminium phosphates, which seem to be more effective in acidic soil of Assam^15^. Again, plants are more vulnerable to pathogens during abiotic stress. Moreover, a solution to provide cross-protection against phytopathogens during abiotic stress is always appreciable. The members of the consortium were also efficient producers of bacterial siderophores. The siderophores play a significant role in the biological availability of iron to plant roots^55^. Low molecular mass siderophores can bind to most of the iron available in the rhizosphere with very high avidity, which inhibits the proliferation of fungal pathogens in host plant roots due to lack of available iron^56,57^. Earlier report has demonstrated the activity of siderophores overproducing mutants in protecting the plants against heavy metal stress^58^. Further, the bacterial strains were efficient producers of HCN. HCN-producing bacteria are antagonistically active against different microorganisms. The HCN may provide protection against pathogenic entry in plants during stressful conditions. Similarly, the bacterial HCN can induce systemic resistance in plants by acting as extracellular signals, subsequently triggering a series of internal processes. Ultimately, the signal translocated is perceived by plant cells which activate cellular defense mechanisms. Hence, the consortium has additional advantages for protecting plants against phytopathogen attacks during abiotic stress. Thus, apart from ACC deaminase activity, multiple PGP traits of the selected strains also may have a cumulative effect in plant growth promotion under drought stressed environment.

Exposure to ACC deaminase positive bacteria has successfully benefitted the host plants by various biochemical and physiological modifications as well. Firstly, successful plant seedling establishment is one of the major concerns for the survival of crop plants during stress conditions. Our tested bacterial consortium showed 100% germination of the tested plant seeds, demonstrating their high performance in plant growth and development during the presence of environmental stressors. Additionally, bacterial priming enhanced the ROS scavenging enzyme activity in stress-induced plants in comparison to the uninoculated stressed plants, showing consistent coalition with previous findings^27,28,59,60^. In principle, the increase of ROS scavengers in host plants is recognized as an important parameter for drought stress alleviation by the microorganism. The level of CAT and POD increased significantly (*p* = 0.05) in consortium-treated stress-affected plants in comparison to the negative controls. The increase in activity of ROS scavenging enzymes (i.e., CAT and POD) provided the protective mechanism in stress-exposed experimental plants by detoxifying the reactive hydrogen peroxide (H_2_O_2_), hydrogen radical (•OH) and singlet oxygen (^1^O_2_), as stated by Kohler *et al*.^28^. One of the major early responses to water stress in plants is a decrease in photosynthetic efficiency. In general, a significant reduction of photosynthesis is observed in plants during drought stress, which thereby decreases energy production and metabolite accumulation. Inoculation of garden pea and black gram plants with ACC deaminase positive consortium partially eliminated the deleterious water stress effects on growth by maintaining the chlorophyll content, which was also noted in previous reports^61,62^. This revealed the higher photosynthetic activity of the consortium-treated plants compared to the negative controls. Furthermore, bacterial inoculation showed a direct effect on proline and phenolic compounds accumulation. Plant cellular osmolytes, like proline and phenolics, are important determinants of plant response to environmental stresses^63^. There is a correlation between increases in osmolyte accumulation and decreases in cellular osmotic potential. The decrease in cellular osmotic potential helps maintain adequate water absorption from drying soil, which thereby increases cell turgor pressure, improving the physiological activity of plants, even during prolonged water deficit^64–66^. Later on, the osmolytes can also act as molecular chaperones, stabilizing the cellular structure of proteins, and they can defend host cell walls by strengthening the exodermis and several cortical cell layers^67^. Our experimental results showed a direct correlation between bacterial inoculation and osmolyte accumulation in treated plants, which could also be an important bacterial determinant for drought stress alleviation in the tested host plants.

## Conclusion

The isolation and characterization of stress-tolerant rhizobacteria are not only essential for understanding their ecological role in the rhizosphere but also their utilization in eco-friendly and sustainable agro-technologies. The overall study has established the combined action of *O*. *pseudogrignonense* RJ12, *Pseudomonas* sp. RJ15, and *B*. *subtilis* RJ46 towards the alleviation of water stress in black gram and garden pea plants. The inoculation of black gram and garden pea plants with consortium resulted in higher seed germination rates, enhanced root length elongation, increased synthesis of total leaf chlorophyll, and accelerated production of antioxidant enzymes and cellular osmolytes. Also, down-regulation of the *ACO* gene transcript was observed upon consortium inoculation in the stressed plants. Besides these mechanisms, the inherent PGP traits of individual bacteria may provide an indirect mechanism for water stress alleviation in the tested plants by providing sufficient phosphate, iron, available nitrogen, and cross-protection against pathogen entry. Thus, the use of such microbial consortium/consortia, which can induce drought stress tolerance and also enhance plant growth and development during normal condition, might be very much beneficial for sustainable agriculture^68^. The integrative application of such consortium having the characteristics of a potential biotic and abiotic stress suppressor might appear to be a very effective strategy for drought stress alleviation in other crops as well. Moreover, the bacterial strain and their consortium formulation require further field evaluation and validation before being confirmed as bio-inoculants to combat various abiotic stresses in the acidic soil based agro-ecosystems of Northeast India.

## Methods

### Soil sampling, isolation of ACC deaminase producing rhizobacteria and screening for osmotic stress tolerance

Rhizosphere soil samples were collected from drought-affected and normally-irrigated agricultural plots of Jorhat district, Assam (26° 45’ 0” North and 94° 13’ 0” East of Northeast India with an average altitude of 116 ma. s. l. and warm-to-temperate climatic conditions). The monsoon months (June to October) receive heavy rainfall, with an average of 412 mm; however, rainfall is scanty during winter (November to February), with an average of 15 mm (Metallurgical Department, Govt. Assam, India). The soils of the selected agricultural field were vertisol type^69^ and clay loam in texture with pH 3 to 5.5. The samplings were carried out during the month of November 2013–2014 and 2014–2015. A total of Fifty-two soil samples were collected aseptically from roots of five different vegetable crops (*Brassica juncea* L., *Phaseolus vulgaris* L., *Pisum sativum* L., *Brassica oleracea* L. and *Vigna mungo* L.). For each crop, ten to twelve plants were randomly selected for rhizosphere soil sampling. The root-associated soil samples were collected during plant growing season as a 15 cm^2^ by 30 cm depth lump. After collection, the roots were shaken vigorously by hand for 10 minutes to remove the loosely-adhering soil particles. The soil particles that were tightly adhered to the roots were then scraped with a brush and tweezers, transferred into separate sterile Hi-dispo Bags (HiMedia, Mumbai, India) and immediately transported to the laboratory by an air-conditioned sampling van at room temperature. Soil suspensions in phosphate saline were spread on a Dworkin and Foster (DF) minimal salt medium with 3 mM ACC as the sole source of nitrogen for selective isolation of ACC-deaminase-producing-rhizobacteria^70^. The pH of the medium was adjusted at the range of 3 to 5.5. Colony PCR with the degenerate primers DegACC5′ (5′-GGBGGVAAYAARMYVMGSAAGCTYGA) and DegACC3′ (5′-TTDCCHKYRTANACBGGRTC) was carried out to amplify partial *acdS* gene for better detection of ACC deaminase positive strain, as mentioned earlier^71^. Bacterial osmotic stress tolerance was checked by monitoring their growth curve under different water potentials (−0.05, −0.15, −0.30, −0.49, and −0.73 MPa). The osmotic stress condition was developed in the Nutrient broth (NB) growth medium by adding the required amount of polyethylene glycol (PEG 6000) as described by Michel and Kaufmann 1973^72^. One millilitre of overnight grown bacterial culture (1 × 10^8^ CFU ml^−1^) was inoculated to the PEG supplemented NB and incubated at 30 ± 2 °C for 24 hours with a continuous agitation of 120 rpm. Bacterial growth was monitored colourimetrically by measuring the absorption spectra at 600 nm as a function of time using Specord 200 (Analytik Zena, Germany). Out of 70 ACC deaminase producers, only three isolates – i.e., RJ12, RJ15 (inhabitants of *Vigna mungo* L. rhizosphere), and RJ46 (inhabitant of *Pisum sativum* L. Rhizosphere) showed vigorous growth at high osmotic stress conditions (−0.30, −0.49, and −0.73 MPa) (Supplementary Material Table S1). Therefore, these three isolates were further considered in the rest of the study. Furthermore, the growth of the isolates was checked in a varied acidic pH range (3 to 5.5) and observed vigorous growth in the pH ranges with the maximum at 4.5 (Supplementary Material Table S2).

### Phenotypic and biochemical characterization of bacterial isolates

The morphology of the three isolates was examined using gram staining and light microscopy. Further biochemical characterization was carried out according to Bergey’s Manual of Determinative Bacteriology^73^.

### Identification of bacterial isolates

The three selected osmotic stress-resistant bacteria strains were identified up to the genus/species level by 16S rRNA signature sequencing. Purified bacterial genomic DNA was taken as the template to amplify 16S rRNA signature sequence with bacterial universal primer 27 F and 1492 R. The amplicons (approx. 1450 bp) were purified using GeNeiPure™ Quick PCR purification kit and sequence were determined by fluorescent terminators (Big Dye, Applied Biosystems) run in an Applied Biosystems ABI prism-automated DNA sequencer (3130 × l). The partial 16S rRNA sequences were compared with NCBI GenBank database using the online software BLASTN. The trimmed 16S rRNA sequences were submitted further in NCBI gene bank and sequence IDs retrieved.

### Quantification of ACC deaminase and other PGP traits

The screening and quantification of *in vitro* PGP traits of the selected bacterial strains were performed in bacterial strains under both normal and osmotic stress conditions. The required amount of PEG was added to develop osmotic stress (−0.73 MPa) in the growth mediums for quantification of the PGP traits under stress. Moreover, the same standard protocols were used for the quantification of PGP traits of the bacterial strains growing under normal and osmotic stress conditions. The quantitative estimation of ACC deaminase was carried out as mentioned by Honma and Shimomura^74^. The three bacterial isolates were grown in a DF minimal broth (pH 4.5) supplemented with 10 µg of ACC (Sigma-Aldrich). After 48 hours of incubation, colourimetric estimations for enzyme activity were carried out and expressed in micromoles of α-ketobutyrate produced per milligram of cellular protein per hour (µmol mg^−1^ h^−1^). The production of an IAA-like molecule was carried out as described by Gordon and Weber^75^. The bacterial strains were inoculated in a DF salts minimal medium with L-tryptophan of different concentrations (0, 50, 100, 200 and 500 µg ml^−1^). The 48-hours-old bacterial cultures were harvested by centrifugation (4000 × *g* for 20 minutes at 4 °C). A preliminary screening of indole production was performed by mixing the supernatant with Salkowski’s reagent (50 ml, 35% perchloric acid and 1 ml 0.5 M FeCl_3_) in a ratio of 1:4 (supernatant: reagent) at room temperature (28 °C) for 20 minutes. The development of a pink colour indicated the production of indoles. Indole production was quantified by spectrophotometric absorption (Specord 200, Analytik Jena, Germany) at 535 nm with three replications. A standard curve was prepared by using pure IAA (Sigma Aldrich, USA). The phosphate solubilisation efficiency was monitored by aluminium-phosphate-supplemented modified Pikovskaya agar, as tricalcium phosphate (TCP) has been reported to be an unreliable and relatively weak factor in determining the solubilization of inorganic phosphate in the acidic soil of Assam^15,76^. Further, the quantitative estimation of phosphatase was carried out^77^. Nitrogen fixation, HCN production, and siderophore production were monitored by previous standard protocols^78–80^. Moreover, the compatibility of the three rhizobacterial strains with each other was tested by dual culture plate assay on nutrient agar and agar well diffusion method^81^.

### Bacterial inoculum preparation

The cells of overnight-grown bacteria (1 × 10^8^ CFU ml^−1^) were harvested by centrifugation (4500 rpm for 20 minutes). The harvested cells were washed twice with 60 mM phosphate saline buffer and resuspended thereof. An optical density of 0.5 at 535 nm was achieved to maintain the uniform cell density of 1 × 10^8^ CFU ml^−1^. For consortium (either any two bacteria or mixed suspension of all three), the cell suspensions were mixed at 1:1 or 1:1:1 ratio.

### Effect of selected isolates on plant growth promotion

The PGP efficiency of the bacterial strains was performed using standard roll towel method^82^. The individual effect of the selected isolates (RJ12, RJ15, and RJ46), a mixture of any two isolates (RJ12 + RJ15, RJ12 + RJ46, RJ15 + RJ46), and the combination of all isolates (consortium) on seed germination and seedling vigor were determined. The black gram (var PU 40) and garden pea (var Goldie) seeds (50 of each) were surface-sterilized with 70% alcohol and 1% sodium hypochlorite and inoculated with 10 ml bacterial inoculum (1 × 10^8^ CFU ml^−1^) containing 0.1% of carboxymethyl cellulose (CMC) as an adhesive agent. After incubation at room temperature for 2–3 hours, the seeds were dried with sterile blotting paper. Surface sterilized uninoculated seeds were considered as the control group. Both inoculated and control seeds were seeded in Hoagland solutions with polyethylene glycol (PEG-6000) and incubated at 25 ± 2 °C in a plant growth chamber (Fitotron, Weiss Technik, UK). The temperature was maintained at 35 °C and 25 °C (day and night) with a relative humidity of 60%. The PEG-6000 was added to develop artificial stress in the Hoagland solution. After 7 days of incubation, seed germination percentage, root length, shoots length, and vigor indexes (VI) were calculated. The VIs was calculated by the formula, VI = % of seed germination × (root length + shoot length)^83^. The whole experiment was repeated five times and carried out with five replications individually for each treatment.

### Growth promotion under osmotic stress (drought stress) condition

The surface sterilized black gram and garden pea plants were treated with bacterial inoculums as mentioned in the earlier section. Averages of 10 seeds/pot were sown in earthen pots containing a sterile soil mixture (clay loam/sand/cow dung at 1:1:1 w/w/w ratio). Seedlings were grown in a greenhouse with 28/20 °C day/night temperatures and ~70% relative humidity under conditions of a 16/8 hours light/dark cycle (approx.). After 10 days of seedling growth, plants were divided into the following categories with five replications of each, viz. (1) uninoculated watered plants as positive control; (2) individual bacteria-inoculated plants under water stress; (3) individual bacteria-inoculated under normal watered condition; (4) combined inoculation of RJ12 and RJ15 under water stress and normal watered conditions, RJ12 + RJ15; (5) combined inoculation of RJ12 and RJ46 under water stress and normal watered conditions, RJ12 + RJ46; (6) combined inoculation of RJ15 and RJ46 under water stress and normal watered condition, RJ15 + RJ46; (7) combined inoculation of all three isolates (RJ12 + RJ15 + RJ46) under water stress; (8) combined inoculation of all three isolates (RJ12 + RJ15 + RJ46) with a normal water supply, and (9) uninoculated plants under drought stress as negative control. The osmotic stress in the pots was artificially induced by irrigating the pot with a PEG-6000 nutrient solution. The concentration of PEG-6000 (g/L of water) was determined using the equation of Michel and Kaufmann^72^.

The osmotic potential of the stress-induced pots was gradually decreased at a rate of -0.04 MPa/day. On the twenty-fifth day of sowing (15 days after the stress induction), the osmotic pressure reached 0.51 MPa. The soil moisture content on the twenty-fifth day was 20% in the negative control and bacteria-inoculated stress-induced plants, and the same condition was maintained up to the forty-fifth day of plant growth. The soil moisture content was determined using 5TE soil moisture sensors (Decagon Devices, Inc., Pullman, WA, USA). The pH of the sterile soil mixture was maintained at 4.5 by watering the plants with leftover (cold) coffee, diluted 50–50 with water. The pH of the soil mixture was measured by a pre-calibrated pH electrode (Mettler Toledo, USA) in 1:5 suspensions of soil and water^84^.

### Morphological and physiological characterization of the experimental plants

The plants were harvested randomly on 45^th^ day (5 plantlets/replicate/treatment, i.e., a total of 25 plantlets per treatment). The harvested plants were further studied for any changes in morphological parameters, such as shoot length, root length, and dry weight. The root water content (RWC) of leaves was determined from the 25 randomly collected plantlets of each treatment using the formula RWC (%) = (Fresh weight − Dry weight/completely turgid weight − Dry weight) × 100^85^. Total chlorophyll content was estimated from two grams of randomly collected leaf samples of each treatment via the standard protocol^86^. Root vigor (expressed in terms of root recovery intension), was also measured according to the triphenyltetrazolium chloride (TTC) method^87^.

### Biochemical characterization

Biochemical characterizations of the experimental plants (bacteria treated, positive control, and negative control) were started on the 17^th^ day after sowing with 7-day intervals up to the 45^th^ day of plant growth. The leaf samples were collected randomly from twenty plantlets of each treatment for further biochemical characterization.

Two grams of fresh leaves were homogenized in 2 ml of a 50 mM ice-cold phosphate buffer (pH 6.0) with pre-chilled mortar and pestle. The homogenate was centrifuged at 15000 × g for 15 minutes at 4 °C. The supernatant was used for enzyme assays. The protein concentration was determined according to the Bradford method using bovine serum albumin (BSA) as standard^88^. The reaction mixture for the POD assay contained a potassium phosphate buffer (160 μl, 100 mM, pH 6.0), an H_2_O_2_ solution (80 μl, 0.5% w/w) and a pyrogallol solution (160 μl, 5% w/v) making the final volume 1.5 ml. Fifty microlitre enzyme extracts were added to the assay solution. The reaction was monitored at 420 nm after 3 minutes of reaction, and the activity was expressed in terms of U mg^−1^ protein with five replicates^89^. CAT activity was measured according to Beer and Sizer (1952), with minor modifications^90^. The reaction mixture consisted of a 100 mmol l^−1^ phosphate buffer (pH 7.0), 0.1 mmol l^−1^ EDTA, 20 mmol l^−1^ H_2_O_2_, and 20 μl enzyme extract. The reaction was started by the addition of 20 µl enzyme extract. After 3 minutes of enzymatic reaction, the decrease of H_2_O_2_ was monitored at 240 nm and quantified by its molar extinction coefficient (36 M^−1^cm^−1^), and the results were expressed as units mg^−1^ protein (U = 1 mM of H_2_O_2_ reduction min^−1^ mg^−1^ protein) with 5 replicates. The total phenolics and proline content were measured with standard protocols and expressed in mg g^−1^, fresh weight, and µmoles g^−1^ fresh weight, respectively, with 5 replicates^91,92^.

### Extraction and measurement of ACC

The ACC contents in the roots of 25 randomly collected plantlets on the forty-fifth day of plant growth were extracted and analyzed from different treatment conditions (uninoculated watered plants as a positive control, consortium-inoculated plants with drought stress induction, uninoculated plants under drought stress as a negative control, and consortium-inoculated with sufficient water supply)^93^. Root apexes were crushed in liquid nitrogen, followed by homogenization in 80% ethanol at 55 °C for 10 to 15 minutes. After centrifugation (10000 × *g* for 10 minutes), supernatants of same samples were evaporated to dryness under vacuum at 55 °C. The final products were suspended in distilled water. Further, the amounts of extracted ACC were quantified indirectly by converting ACC to ethylene. The evolved ethylene was measured by gas chromatography. The whole experiment was repeated three times with five replications for each treatment.

### RNA isolation and two steps real-time PCR

The total RNA extraction from the leaf and root samples of normal plants, as well as of plants that experienced stress for 45 days, were extracted by the RNeasy plant mini kit (Qiagen, Leusden, The Netherlands) and immediately reverse-transcribed to cDNA with a 2 × Verso cDNA synthesis kit (Thermo Scientific, USA) using random hexamers as per the manufacturer’s instruction. A quantitative amplification reaction for reference and target genes were carried out in a 96-well StepOnePlus^TM^ Real-Time PCR System (Applied Biosystems, USA) using a Thermo DYNAMOTM 4 C SYBR Green qPCR master mix (Thermo Scientific, USA). cDNAs were replaced by sterile water for no-template control reaction. Fifteen nanograms (15ng) of cDNAs were used for the relative expression analysis. The reaction conditions were set as follows: 10 min. at 42 °C; 10 min. at 95 °C; 40 cycles of cDNA amplification for 15 s at 95 °C, 30 s at 60 °C, and 30 s at 72 °C with fluorescent signal recording. At the end, a final step of 15 s at 95 °C, and of 1 min. at 60 °C and fluorescence measured at each 0.7 °C variation (from 60 °C to 95 °C) was included to obtain the melting curve. Four reference genes, i.e. *Act11*, the Ubiquitin-conjugating enzyme (*Ubq*), Tubulin β-9 (*β-Tub9*), and 18S rRNA were used for the proper normalization of real-time qPCR reaction. Two important stress ethylene regulatory genes ACC oxidase (*ACO*) and ACC synthase (*ACS*) were selected as target genes for the experiment. The sequences of the genes studied were obtained from NCBI GenBank and the primers were designed with the aid of the OLIGO software (version 5.0; Molecular Biology Insights). The sequences, Genebank accession ID, and other parameters of primers have been listed in Table 5.

**Table 5:** Primers sequences and other properties used in real time PCR expression analysis.

Target genes	Genebank ID	Target plants	Forward sequence (5′-3′)	Reverse sequence (5′-3′)	Tm (°C)	Product size (bp)	PCR efficiency value (E ± SD)
*ACO*	AB128037.1	GP	CTTGTCCTAAACCGGCACTC	CACTTCGAGTGGATCACCAA	59	179	1.854 ± 0.052
	AM180696.1	BG	TGCTGTGATTTCTCCAGCAC	ACGGCCTTCATAGCTTCAAA	59	150	1.932 ± 0.031
*ACS*	AF016458.1	GP	GGAGGATTCAAACGTGATGG	GGAGGATTCAAACGTGATGG	60	234	2.012 ± 0.061
	M94863.1	BG	TCTGCTGCAGGTTTCATTTG	TGCTCTCCCACCTCTCACTT	60	181	1.883 ± 0.048
*ACT11*	U76192.1	GP	TGAAGCTCCGCTTAACCCTA	ATCGCATGTGGAAGTGCATA	60	207	2.121 ± 0.056
	NM001278957.1	BG	TCCTCTCACCTTGCCTCTGT	TCCAGCCTTAACCATTCCAG	60	151	1.922 ± 0.024
*Ubq*	L29077.1	GP	CCGTATGCTGGAGGTGTTTT	GGATCAGTCAGCAATGAGCA	59	209	1.787 ± 0.047
	CM003604.1	BG	GCTCAAGGATTTGCAGAAGG	TTGGTGGCTTGAAGGGATAG	60	170	1.865 ± 0.032
*β- Tub9*	FE676365.1	GP	GGATCTCGAACCTGGAACAA	CCAAGACGGAATCGATGAGT	60	155	1.951 ± 0.027
	X60216.1	BG	CCGTTGTGGAGCCTTACAAT	TCCACTCATGGTGGCAGATA	60	170	1.843 ± 0.043
18S rRNA	AH001723.2	GP	CATGATAACTCGTCGGATCG	CGTTTCTCAGGCTCCATCTC	59	220	2.011 ± 0.027
	AH001765.2	BG	AGCGGATGTTGCTTTTAGGA	GCACCACCACCCATAGAATC	59	225	1.943 ± 0.048

Table key: BG - black gram, GP - garden pea, Tm- melting temperature.

### Toxicity test

All three bacterial strains underwent toxicity tests at APT Testing and Research Private Ltd., Pune, India, to investigate acute oral toxicity/pathogenicity.

### Data analyses

A one-way ANOVA, followed by Tukey’s test, was conducted to analyze the data sets obtained from the quantitative estimation of PGP traits (Table 1). Student’s t-test was used to analyze the data of seed germination/vigor index experiments (Table 2). However, the rest of the greenhouse experiments and real-time PCR generated results were analyzed by a two-way ANOVA, considering water supply and bacteria inoculation as two independent variables, followed by Tukey’s post-test for each treatment using SPSS software (ver. 10.1, SPSS Inc., www.spss.com). The significance level for all analyses was *p* = 0.05. In real-time qPCR experiments, ten-fold serial dilution of cDNA curves was used to calculate the amplification efficiency for all genes using the formula E = 10^(−1/slope)^^94^. The threshold cycle (CT) was compared with the log_10_ relative copy number of the sample from a dilution series. The CT values and log copy numbers of cDNAs for all the genes maintained a linear relationship having a range of correlation coefficient (R^2^) from 0.95 to 0.99, indicating a proportionate change in CT values related to the serial dilution of the samples. The E-value ranged from 1.787 to 2.121, indicating the efficient amplification near the theoretical optimum level of 2^95^. The relative expression levels obtained for target genes were compared when the candidate normalizer genes were used individually. Then, the best combination was obtained by geNorm software^96^. The expression level calculated by the formula 2^−ΔΔCt^ represents the x-fold difference from the calibrator.

## Electronic supplementary material

Supplementary Information
