# Author Correction: Alleviation of drought stress in pulse crops with ACC deaminase producing rhizobacteria isolated from acidic soil of Northeast India

**DOI:** 10.1038/s41598-018-25174-5

**Published:** 2018-04-30

**Authors:** Juthika Saikia, Rupak K. Sarma, Rajashree Dhandia, Archana Yadav, Rupjyoti Bharali, Vijai K. Gupta, Ratul Saikia

**Affiliations:** 1 Biotechnology Group, Biological Sciences and Technology Division, CSIR-North East Institute of Science and Technology, Jorhat, 785006 Assam India; 2 Department of Biotechnology, Gauhati University, Guwahati, 781014 Assam India; 3 Department of Chemistry and Biotechnology, ERA Chair of Green Chemistry, Tallinn University of Technology, Tallinn, 12618 Estonia

Correction to: *Scientific Reports* 10.1038/s41598-018-21921-w, published online 23 February 2018

In this Article, Vijai K. Gupta is incorrectly listed as being affiliated with ‘Applied Biology and Biopharmaceutical Science, School of Science & Computing, Galway-Mayo Institute of Technology, Galway, Ireland’

The correct affiliation is listed below:

‘Department of Chemistry and Biotechnology, ERA Chair of Green Chemistry, Tallinn University of Technology, Tallinn 12618, Estonia’

This error has now been corrected in the PDF and HTML versions of the Article, and in the Supplementary Information file.

