# Supplementary material for: Alleviation of drought stress in pulse crops with ACC deaminase producing rhizobacteria isolated from acidic soil of Northeast India
Source: Sci Rep. 2018 Feb 23;8:3560. doi: 10.1038/s41598-018-21921-w (PMC5824784; doi:10.1038/s41598-018-21921-w)
Supplement: Supplementary file 1 — Supplementary Information [file 41598_2018_21921_MOESM1_ESM.docx]

**Alleviation of drought stress in pulse crops with ACC deaminase producing rhizobacteria isolated from acidic soil of Northeast India**

**Juthika Saikia^1^, Rupak K. Sarma^1^, Rajashree Dhandia^1^, Archana Yadav^1^, Rupjyoti Bharali^2^, Vijai K. Gupta^3^, Ratul Saikia^1^**^*^

*^1^Biotechnology Group, Biological Sciences and Technology Division, CSIR-North East Institute of Science and Technology, Jorhat 785006, Assam, India*

*^2^Department of Biotechnology, Gauhati University, Guwahati 781014, Assam, India*

*^3^Department of Chemistry and Biotechnology, ERA Chair of Green Chemistry, Tallinn University of Technology, Tallinn 12618, Estonia*

*First, second and third authors equally contributed to the paper*

**Corresponding author.* Biotechnology Group, Biological Sciences and Technology Division, CSIR-North East Institute of Science and Technology, Jorhat 785006, Assam, India

*E-mail addresses*: rsaikia19@gmail.com, [ratul19saikia@gmail.com](mailto:ratul19saikia@gmail.com) (R. Saikia)

**Table S1**

Optical density (OD) of the three rhizobacterial isolates at 600 nm after 24 hours of growth under different osmotic stress condition considering normal growth in nutrient broth (NB) as control.

| Isolate | Control | -0.05MPa | -0.15MPa | -0.30MPa | -0.49MPa | -0.73MPa |
| --- | --- | --- | --- | --- | --- | --- |
| *Ochrobactrum pseudogrignonense* RJ12 | 2.70±0.16 | 2.53±0.12 | 1.75±0.05 | 0.86±0.01 | 0.77±0.01 | 0.28±0.01 |
| *Pseudomonas* sp. RJ15 | 2.77±0.12 | 2.70±0.08 | 1.86±0.12 | 0.96±0.01 | 0.88±0.01 | 0.33±0.01 |
| *Bacillus subtilis* RJ46 | 2.60±0.22 | 1.87±0.05 | 1.65±0.04 | 0.64±0.01 | 0.74±0.03 | 0.26±0.01 |
|  |  |  |  |  |  |  |

**Table S2**

Optical density (OD) of the three rhizobacterial isolates at 600 nm after 24 hours of growth under different acidic pH ranges

| Isolate | pH 3 | pH3.5 | pH4 | pH4.5 | pH5 | pH5.5 |
| --- | --- | --- | --- | --- | --- | --- |
| *Ochrobactrum pseudogrignonense* RJ12 | 1.67±0.32 | 1.92±0.16 | 2.54±0.13 | 2.86±0.23 | 2.7±0.33 | 2.71±0.25 |
| *Pseudomonas* sp. RJ15 | 1.52±0.44 | 1.83±0.21 | 2.51±0.25 | 2.72±0.41 | 2.64±0.53 | 2.5±0.17 |
| *Bacillus subtilis* RJ46 | 1.56±0.27 | 1.77±0.33 | 2.48±0.37 | 2.70±0.36 | 2.6±0.14 | 2.51±0.24 |

**Table S3**

| Treatments | Root length  (cm) | | Shoot length  (cm) | | Dry weight  (g) | | Relative water content (%) | |
| --- | --- | --- | --- | --- | --- | --- | --- | --- |
|  | BG | GP | BG | GP | BG | GP | BG | GP |
| Inoculated with RJ12+RJ15under stress | 7.3±1 | 7.23±1.2 | 8.4±0.23 | 7.3±1.02 | 35±2 | 42±1.6 | 59±2 | 59±2 |
| Inoculated with RJ12+RJ15  and sufficient water supply | 3.65±0.98 | 2.89±0.43 | 10.6±1.2 | 11.2±0.65 | 51±1.8 | 58±2.1 | 68±2.1 | 67±1.9 |
| Inoculated with RJ12+RJ46  under stress | 10.3±1.5 | 7.74±0.82 | 8.6±1 | 7.6±1 | 39±1.6 | 47±1.8 | 65±2.5 | 71±1.6 |
| Inoculated with RJ12+RJ46  and sufficient water supply | 3.21±0.67 | 3±0.12 | 12.2±1.5 | 13±1.5 | 54±2.1 | 64±2 | 69±1.9 | 78±2.6 |
| Inoculated with RJ15+RJ46under stress | 8±0.91 | 7±1.2 | 8±0.4 | 8.6±0.2 | 28±1.5 | 45±1 | 55±1.7 | 62±1.8 |
| Inoculated with RJ15+RJ46 and sufficient water supply | 2.69±0.2 | 2.77±0.32 | 10.7±2 | 12.1±1.6 | 46±2 | 55±1.7 | 64±2.2 | 69±1 |

Effect of bacterial inoculation on plant growth promotion.

BG - black Gram, GP - garden pea

**Figure S1.** Dual culture plate assay to check the compatibility of bacterial isolates with one another. The assay did not show any antagonistic activity of the isolates among them.

**
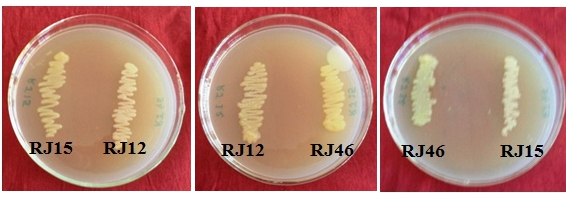
**
